# The Important Function of Mediator Complex in Controlling the Developmental Transitions in Plants

**DOI:** 10.3390/ijms21082733

**Published:** 2020-04-15

**Authors:** Lingjie Zhang, Changkui Guo

**Affiliations:** State Key Laboratory of Subtropical Silviculture, School of Agriculture and Food Sciences, Zhejiang Agriculture and Forestry University, Hangzhou 311300, China; 18763821796@163.com

**Keywords:** mediator complex, vegetative phase change, floral transition, plants

## Abstract

Developmental transitions in plants are tightly associated with changes in the transcriptional regulation of gene expression. One of the most important regulations is conferred by cofactors of RNA polymerase II including the mediator complex, a large complex with a modular organization. The mediator complex recruits transcription factors to bind to the specific sites of genes including protein-coding genes and non-coding RNA genes to promote or repress the transcription initiation and elongation using a protein-protein interaction module. Mediator complex subunits have been isolated and identified in plants and the function of most mediator subunits in whole life cycle plants have been revealed. Studies have shown that the Mediator complex is indispensable for the regulation of plant developmental transitions by recruiting age-, flowering-, or hormone-related transcription factors. Here, we first overviewed the Mediator subunits in plants, and then we summarized the specific Mediator subunits involved in developmental transitions, including vegetative phase change and floral transition. Finally, we proposed the future directions to further explore their roles in plants. The link between Mediator subunits and developmental transitions implies the necessity to explore targets of this complex as a potential application in developing high quality crop varieties.

## 1. Introduction

**Figure 1:**
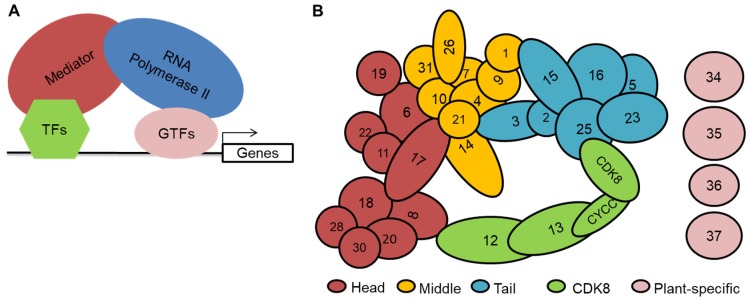
Working model and subunit composition of the Mediator complex. (**A**), Working model of the Mediator complex-dependent transcriptional regulation. Transcription factors bind to the promoter of genes and then deliver the signal to RNA polymerase II by interacting with specific Mediator subunit. TFs, transcription factors; GTFs, general transcription factors. (**B**), The illustrations represent the modular organization of the Mediator complex in Arabidopsis based on the interaction map of Mediator complex and the genetic interaction. Mediator comprises four modules, including Head (red), Middle (yellow), Tail (blue), and CDK8 (green), and four plant specific subunits (pink).

As sessile organisms, plants are able to adapt to changes around them, including decrease of nutrient and water content, increase of salt content in soil, or changeable temperature and light intensity [1]. At the molecular level, when plants perceive dangerous signals from the environment, the expression of numerous immune-related genes is activated to respond to the harsh environment stresses. The alterations in the regulation of gene expression are controlled by lots of cofactors of RNA polymerase II (RNAP II or Pol II), including the Mediator complex. The regulation of Mediator complex mainly occurs at the transcriptional level, and it is also conserved in eukaryotes. It first recruits transcription factors through protein-protein interaction, and then those transcription factors bind to the specific sites of target genes. The other part of Pol II transcription complex works to act in the transcription process to activate or repress the target genes in plants [2,3]. During this process, the Mediator complex is considered as the most important linker between Pol II and transcription factors (Figure 1A). The Mediator also functions in stabilizing the transcription initiation complex, DNA bending, transcription elongation, transcription termination, and chromatin-associated DNA remodeling [4].

The Mediator complex is enormous in size and complex in its composition. It was initially discovered in yeast (*Saccharomyces cerevisiae*) in 1990 [5], and 17 years later, the complex was identified in the model plant, *Arabidopsis thaliana* [6]. Thereafter, the Mediator complex from other eukaryotes was analyzed by bioinformatical methods, X-ray crystallography, cryo-electron microscopy (cryo-EM), and cross-linking mass spectrometry (CXMS) and so on. Most of their subunits have counterparts in yeast. Overall, Mediator has 20~38 subunits with a conserved core set, and is divided into four modules, including the head module, the middle module, the tail module, and the CDK8 kinase module. Each module governs distinct functions in transcription. In yeast, the head module, mainly interacting with RNAP II, contains MED6, MED8, MED11, MED17, MED18, MED20, and MED22; the tail module consists of MED2, MED3, MED5, MED14, MED15, and MED16, and recruits gene-specific transcription factors; the middle module has seven subunits, including MED1, MED4, MED7, MED9, MED10, MED19, MED21, and MED31, and its function is to link the tail and the head for transmitting signal; the CDK8 (Cyclin dependent kinase 8) kinase module, acting as transcription repressor, is composed of MED12, MED13, CDK8, and CYCC (C-type cyclin) [4,7,8]. The cryo-electron microscopy map of *Schizosaccharomyces pombe* Mediator shows that the MED14 subunit serves as a central backbone that connects the head, middle and tail modules. The flexibility of MED14 promotes the large-scale rearrangements for the interaction with RNA polymerase II [9]. MED13 functions to integrate the CDK8 module with the whole complex, and the contact between the head module and the middle module relies on the contact between MED6, MED17 and MED14 [10,11]. Therefore, the stabilization and flexibility of the Mediator complex are required for RNAP II interaction as well as their functions.

The discovery and the functional study of Mediators in *Arabidopsis* and crop plants have helped us to understand many important roles of Mediators in regulating plant development and response to stresses. To provide novel insight into the application of Mediators in crop breeding, we summarized the function of the Mediator complex in regulating developmental transitions, including vegetative phase change and floral transition, and particularly we emphasized how Mediator subunits function in the regulation of developmental transitions.

## 2. Overview of the Mediator Complex in Plants

The first plant Mediator complex was purified from *Arabidopsis thaliana* in 2007 by column chromatography and immunoprecipitation methods based on the Mediator sequence of yeast [6]. The plant Mediator subunits share very low homology with those from other species; and originally six A. thaliana-specific Mediator subunits, including MED32, MED33, MED34, MED35, MED36 and MED37, are isolated by immunoprecipitation and reverse-phase liquid chromatography-electrospray ionization-tandem mass spectrometry (LC-ESI-MS/MS). Moreover, bioinformatical results indicate that the Arabidopsis subunits MED27, MED32 and MED33 were the homologs of the yeast/metazoan subunits MED3/MED27, MED2/MED29, and MED5/MED24, respectively [6]. Therefore, only four subunits (MED34, MED35, MED36 and MED37) are considered as the plant-specific Mediator subunits (Figure 1B). The kinase module subunits, including MED12, MED13, CDK8 and CYCC, could not be purified with other Arabidopsis Mediator subunits; they were only identified by bioinformatics and genetic analyses. To search the lost Mediator subunits in the purified Arabidopsis Mediator database, MED26 with three paralogs in *Arabidopsis* is identified by a conserved TFIIS helical bundle in the N-terminal region. Arabidopsis MED26 possibly interacts with P-TEFb (POSITIVE TRANSCRIPTION ELONGATION FACTOR b) to promote the transcription initiation to elongate [12]. MED1 was the last Mediator subunit to be identified in *Arabidopsis*. In a yeast two-hybrid (Y2H) screen of interactors of CCG (CENTRAL CELL GUIDANCE), CCG-BINDING PROTEIN 1 (CBP1) was found to interact with CCG1. CCG1 has been shown to interact with the general transcription machinery, and to bind to the CTD (C-terminal domain) of Pol II [13]. CBP1 can also interact with MED7 and MED9 in *Arabidopsis*, like that in yeast and human. Therefore, CBP1 is potentially the orthologue of MED1, but its function is diverse in the plant lineage [13]. To date, 34 Mediator subunits (54 homologs) in *Arabidopsis* have been identified using biochemical purification and bioinformatical methods (Table S1). The Mediator complex homologs of rice (*Oryza sativa*) and maize (*Zea mays*) are 55 and 56, respectively; whereas the number of Mediator complex homologs of soybean (*Glycine max*) is 92, much more than that of *Arabidopsis* (Table S1). The main cause is that soybean has a highly duplicated genome. In addition, the plant-specific Mediator subunits have many more paralogs than other subunits (Table S1), suggesting that those subunits possibly perform a specialized function in plants or balance the plant development.

The precise transcriptional regulation of gene expression is required for proper plant growth and development. The Mediator complex acts as an important regulatory hub in controlling the transcriptional regulation of some functional genes. Therefore, where and when the Mediator subunits are expressed is critical for assessing their functional sites and functional stages in growth and development. To answer these questions, the expression of Mediator subunits was analyzed in different tissues and developmental stages in *Arabidopsis* and rice. *AtMED36* is highly expressed in the root, indicating that it possibly participates in the regulation of root-specific genes [14]. In the stem, *AtMED37*, *AtMED11*, *AtMED7*, *AtCDK8* and *AtCYCC* are expressed at higher levels [14]. *OsMED31_1* is expressed more in leaves than in other tissues [12]. *AtMED7*, *AtCYCC* and *AtMED37* are abundant in juvenile leaves; whereas the expression of *AtMED3*, *AtMED4*, *AtMED7*, *AtMED11*, *AtMED15*, *AtMED18*, *AtMED21*, *AtMED37*, *AtCDK8* and *AtCYCC* is moderately higher in adult leaves [14]. The expression of *AtMED18* and *AtMED15* increases with age, and the expression of *AtMED2*, *AtMED8*, *AtMED5*, *AtMED22*, *AtMED31*, *AtMED5*, *AtMED6*, *AtMED17*, *AtMED36*, *AtMED34*, *AtCYCC* and *AtMED37* decreases with age, suggesting that they might function in the age-dependent pathway to regulate developmental transitions [14]. *OsMED21*, *OsMED8* and *OsMED11_1* are highly expressed in panicles, indicating that they possibly involve the early stages of panicle development in rice [12]. *AtMED37*, *AtMED18*, *AtMED22* and *AtMED7* are highly expressed in old flowers, whereas the expression of *AtMED2*, *AtMED4*, *AtMED15*, *AtMED14*, *AtCYCC* and *AtCDK8* is high in young flowers, implying that these Mediator subunits might function in regulating flower development [14].

To determine how the Mediator complex affects transcription initiation, the structure and interaction of the Mediator complex should be clearly understood. Some evidences have shown that the Mediator complex is dynamic and highly flexible, and its composition influences the structure [9,11]. The expression data show that the different levels of Mediator subunits during certain developmental stages or in specific tissues determine the structural Mediator arrangement to execute specific function. The Mediator complex with high-homology sequences (MED domain) (Table S1) has conserved function by interacting with RNAP II. However, most Mediator subunits lack common functional motifs, and only some of them contain conserved protein interaction domains. MED15 in *Arabidopsis* contains a conserved KIX (kinase inducible domain interacting domain) domain for interactions with the transcriptional activators [15]. MED25 contains a von Willenbrand factor type a (vWFA) domain and an activator-interacting (ACID) domain (MED25-ACID). The vWFA domain strengthens the communion between MED25 and other Mediator subunits, and the ACID domain can bind transcription factors [16,17]. These findings indicate that MED sequences have no high identity, but different domains contain similar structures for interacting with their partners. Moreover, the Mediator subunits have a large range of isoelectric points (Table S1). The large variation increases the possibility to interact with other factors to respond to the harsh environment.

To understand the functional mechanism of the Mediator complex in plant development, an interaction map and a probable structural topology of the Arabidopsis Mediator complex were constructed (Figure 1B). Most topology of the Arabidopsis Mediator is similar to that of the yeast, but there are many special interactions detected in *Arabidopsis*. MED19 in yeast and mammal belongs to the middle module, whereas it is the composition of the head module in *Arabidopsis*. Moreover, MED14 and MED26 in Arabidopsis are the part of the middle module (Figure 1B). AtMED14 and AtMED17 are found as the master component of the whole Mediator complex [18]. AtMED6 links the head module with the middle module, and AtMED10 connects the middle with the tail [18]. However, the yeast MED14 is the main linker to connects the head, middle and tail modules [11]. Moreover, AtMED6, AtMED17, AtMED10, AtMED19, AtMED15, and AtMED4 form homodimers as an initial step to control the Mediator function in the plant cell. The full-length of AtMED9, AtMED21 and AtMED25 possesses transactivation property, whereas parts of AtMED4 and AtMED10 display transactivation property [18]. In yeast, MED2, MED3 and MED15 can activate the reporter genes, while AtMED2 and AtMED3 have no transactivation ability in *Arabidopsis* [18]. Therefore, we speculate that the specialized function of the mediator complex in different eukaryotes might be caused by the differences in the interaction map of Mediator subunits.

## 3. Functions in Vegetative Phase Change in Plants

The whole life cycle of *A. thaliana* is divided into four stages, including embryonic stage, juvenile vegetative stage, adult vegetative stage and reproductive stage. The process of transition from the juvenile stage to the adult stage is referred to as vegetative phase change, whereas the process of transition from the adult stage to the reproductive stage is called reproductive phase change or floral transition [19]. The precise developmental phase transitions, including vegetative phase change and floral transition, guarantees plant fitness, survival, and reproductive success.

**Figure 2:**
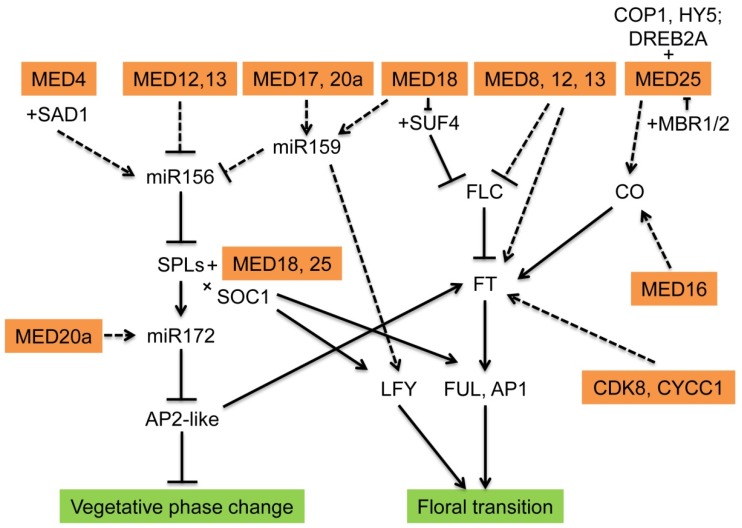
The function of Mediators in developmental transitions. +: Protein-protein interactions; Dashed arrow: indirectly promote; solid arrow: directly promote; and T-bar: restrain. Figure modified from Buendía-Monreal and Gillmor (2016) [7].

In *Arabidopsis*, the juvenile rosette leaves are usually small and round with trichomes only on the adaxial (upper) surface, while adult leaves have increased length-to-width ratios, increased degree of serrations on the leaf margin, and they also produce trichomes on the abaxial (lower) and the adaxial (upper) surfaces. The appearance of abaxial tricomes on rosette leaves is usually used as a morphological marker to distinguish between the juvenile leaves and the adult leaves [20,21,22,23]. Genetic and molecular evidence has indicated that microRNA156 (miR156)-*SQAMOSA PROMOTER BINDING PROTEIN-LIKE* genes (SPLs) plays a master regulatory role in vegetative phase change [24,25,26,27,28]. miR156 acts as a negative regulator in vegetative phase change. Overexpression of miR156 prolongs the juvenile phase, while silencing the expression of miR156 accelerates the juvenile-to-adult transition [27]. miR156 is highly expressed in the juvenile phase, and its expression declines as plants age, while its targets, SPLs, increase with age. Likewise, the expression of another miRNA, miR172, a direct transcriptional target of SPL9 and SPL15 [27,29], increases as plants age to promote vegetative phase change by repressing its targets, including APETALA2 (AP2), TARGET of EAT1 (TOE1), TARGET of EAT2 (TOE2), TARGET of EAT3 (TOE3), SCHLAFMÜTZE (SMZ) and SCHNARCHZAPFEN (SNZ) [27,30,31,32,33,34]. In addition to this intrinsic regulator of vegetative phase change in plants, two phytohormones, gibberellic acid (GA) and jasmonic acid (JA) have also shown to play important roles in this process. GA accelerates vegetative phase change [35], whereas JA prolongs the juvenile stage in rice and maize by promoting the expression of miR156 [36,37]. In plants, the role of Mediator in vegetative phase change has been described previously (Figure 2, Table 1).

Arabidopsis MED12 (CENTER CITY, CCT) and MED13 (GRAND CENTRAL, GCT), belonging to the Mediator CDK8 modules, were first identified as regulators of the timing of early embryogenesis as well as a regulator of seed-to-seedling transition by affecting embryonic gene expression independently of GA [38]. Genetic and molecular results showed that *gct* and *cct* mutants prolong the juvenile phase by increasing the amount of miR156 and reducing the expression of miR172 and *SPL3* [38]. GCT and CCT regulate vegetative phase change in parallel to GA [38]. Sugar represses the miR156 expression in the presence or absence of CCT or GCT, indicating that sugar and the CDK8 module regulate miR156 independently, and act together in a synergistic manner to control vegetative phase change [39]. Moreover, the level of miR159 is similar between *gct* or *cct* and wild type, indicating that GCT and CCT affect vegetative phase change in parallel to miR159 [40]. Rice *super apical dormant 1* (*sad-1*) mutant with late appearance of mid-rib delays the juvenile-to-adult transition, it has high levels of miR156 and low levels of miR172 [41]. *SAD1* encodes the orthologue of RPA34.5, a subunit of RNA polymerase I (Pol I). It interacts with OsMED4 to regulate the expression of Ribosomal RNA, and then the expression of phase transition-related genes, including miR156 and miR172 [41]. The leaves of *med17*, *med18* and *med20a* mutants have shorter petioles, and they are curled downward. The accumulation of miR159, which can repress the miR156 level [40], is lower in *med17*, *med18* and *med20a* mutants than that in wild type [42]. In *med20a* mutant, the miR172 level is reduced compared with wild type. These results suggest that MED17, MED18 and MED20 possibly modulate vegetative phase change by affecting the biogenesis of noncoding RNAs, including miR159 and miR172 [42]. Moreover, MED25 is considered as an integrator of JA-mediated transcriptional activation by interacting with MYC2 (a basic helix–loop–helix transcription factor) and HAC1 (HISTONE ACETYLTRANSFERASE1) [43,44]; and MED25 also couples alternative splicing of *JAZ* (*JASMONATE ZIM-DOMAIN*) genes with fine tuning of JA signaling [45]. Based on the important function of JA signaling in vegetative phase change, whether MED25 plays a role in the regulation of vegetative phase change needs to be studied further.

## 4. Functions in Floral Transition

Floral transition, the transition from the vegetative phase to the reproductive phase, is controlled by at least five signaling pathways, including photoperiod, vernalization, gibberellin, aging, and the autonomous pathway. These flowering signaling pathways converge at some floral integrator genes, including CONSTANS (CO), FLOWERING LOCUS C (FLC), SUPPRESSOR OF OVEREXPRESSION OF CONSTANS 1 (SOC1), FLOWERING LOCUS T (FT), AGAMOUS-LIKE24 (AGL24), LEAFY (LFY), APETALA1 (AP1), SEPALLATA3 (SEP3), FRUITFULL (FUL), SHORT VEGETATIVE PHASE (SVP), miR156, SPLs, and miR172 [24,46,47,48,49]. Most Mediator mutants have late flowering phenotypes, including *med8*, *med12* (*cct*), *med13* (*gct*), *med15*, *med16*, *med17*, *med18*, *med20a*, *med23*, *med25*, and *med30* [7], whereas the *med2* and *med5ab* mutants are early flowering. Some of these Mediators function in multiple flowering pathways (Figure 2, Table 1).

In the head module subunits, MED8, MED17, MED18, MED20, and MED30 positively regulate floral transition. *med8* and *med18* mutants show the late flowering phenotype by elevating the expression of *FLC* and repressing the level of *FT* [50,51,52,53,54]. MED18 regulates flowering time through the vernalization pathway, but not the GA pathway [51]. MED18 recruits SUPPRESSOR OFFRIGIDA 4 (SUF4) to directly bind to the *FLC* promoter to repress *FLC* expression. However, SUF4 and MED18 have contrasting functions in flowering regulation since loss-of-function of *SUF4* leads to early flowering [52]. It is possible that the function of SUF4 in directly promoting *FLC* expression is inhibited in the presence of MED18 [52]. MED18 also functions in floral transition through the age- and GA-dependent pathways. RNAP II and MED18 recruit SPL15-SOC1-REF6 (RELATIVE OF EARLY FLOWERING 6) complex to remove the H3K27m3 of *miR172B* and *FUL* to stimulate their expressions [29]. In addition, MED17, MED18 and MED20 also modulate the production of small and long noncoding RNA production. *med17*, *med18* and *med20a* mutants are smaller than wild type and are late flowering, and their leaves have short petioles and are curled downward [42]. miR172, positive controller of floral transition, is decreased in *med20a* mutant [42]. MED30 promotes flowering by decreasing the expression of *SPL3*, *FT* and *SOC1*, and increasing the level of *FLC* [53].

The tail module subunits play dual roles in flowering regulation. Among them, MED15, MED16, MED23 and MED25 positively regulate floral transition, whereas MED2 and MED5 negatively control flowering time in plants. PFT1 (PHYTOCHROME AND FLOWERING TIME 1), also called MED25, functions in the phyB (phytochrome B) pathway to promote flowering in long-day conditions [54]. MED25 promotes flowering by CO-dependent and -independent mechanisms, and it regulates the expression of *FT* downstream of phyB [54]. MED25 can interact with DREB2A (drought response element protein B) to repress the PhyB-mediated light signaling [17]. Likewise, MED25 cooperates with CONSTITUTIVE PHOTORMORPHOGENIC1 (COP1) in the regulation of light response with the presence of ELONGATED HYPOCOTYL5 (HY5) [55]. Yet, *pft1-2* plants are early flowering in short-day conditions, implying that its role is more complex in short-day conditions [55]. There is a conserved short tandem repeat (STR) in the Arabidopsis PFT1/MED25. The STR encodes an interrupted polyglutamine tract (polyQ). PFT1 functions either as an activator or as a repressor of flowering in a photoperiod-dependent manner based on the length of PFT1 STR [56]. In other words, polyQ is crucial for PFT1’s activity [56]. MED25 also involve the age-dependent pathway. MED25 regulates floral transition downstream of SPL10 genetically. SPL10 recruits MED25 to the promoters of *FUL* and *LFY* to promote their expression to regulate flowering [57]. Moreover, MED25-BINDING RING-H2 PROTEIN1 (MBR1) and MBR2 bind to PFT1 and promote PFT1 degradation through RING-H2-dependent way to regulate the *FT* expression [58].

Arabidopsis *med16* (*sensitive to freezing6*, *sfr6*) mutants are late flowering independently of sucrose supplementation in long days, and they show reduced expression of the circadian clock genes, including *CIRCADIAN CLOCK ASSOCIATED1* (*CCA1*), *GIGANTEA* (*GI*), *TIMING OF CAB1* (*TOC1*), *CO* and *FT*, and increased expression of *FLC* gene [59]. In short-day conditions, the *med15* (*non-recognition-of-BTH4*, *nrb4*) mutants do not produce flowers. When they are transferred to long-day conditions, *nrb4-4* plants bolt with no seeds [60]. These results indicate that MED15 may function in light signaling pathway to control flowering. *med23* plants have slightly more rosette leaves than wild type, indicating that they are late flowering. Most importantly, *MED23* is strongly expressed in the shoot apical meristem, and is co-expressed with floral, or meristem development genes. In addition, floral specification gene *AGAMOUS* (*AG*) is induced in *med23* plants [61]. *med2* and *med5ab* mutants are early flowering [61]. In *med2* mutant, *FLC* is elevated, while the expression of its targets, *SOC1* and *FT*, has no significant change, indicating that FLC might partially requires MED2 to restrain floral transition [61]. Because the effect of *med5ab* on floral transition is subtle, the differently expressed genes do not contain the flowering-related genes; whereas gene ontology (GO) term enrichment analysis shows that GO-term “response to JA (GO:0009753)” is enriched in the *med5ab* mutant, suggesting that MED5 possibly inhibits flowering by the JA pathway [61].

CDK8 module subunits, MED12/CCT/CRYPTIC PRECOCIOUS (CRP), MED13/GCT/MACCHIBOU 2 (MAB2), pea CDK8 and pea CYCC1, promote flowering by involving multiple flowering pathways and at multiple regulatory levels [38,62]. MED12 and MED13 negatively regulate *FLC* expression, and positively modulate *FT* and *TSF* (*TWIN SISTER OF FT*) expression independently of *FLC*; in addition, MED12/CRP acts in part downstream of *FT* to regulate the expression of *SOC1* and *FUL* [38,62]. *gct* and *cct* mutants have the similar sensitivity to GA, indicating that GCT and CCT regulate flowering in parallel to GA [38,62]. *med12* is sufficient to rescue the stunted growth of *ref4-3* (*reduced epidermal fluorescence 4*, *med5*) mutant, whereas *ref4-3 med12* also exhibited a late flowering phenotype, indicating that *ref4-3* and *med12* have synergistic effects on flowering [63]. Mutations in CDK8 and CYCC1 delay flowering, and prolong the reproductive phase in pea (*Pisum sativum* L.) by reducing the expression of *FTa1* and increasing the expression of *LF* (*LATE FLOWERING*), a pea TERMINAL FLOWER 1 (TFL1) co-orthologs [64]. A recent report shows that CDK8 is required for the function of circadian clock transcription factors, including LHY (LATE ELONGATED HYPOCOTYL), CCA1 and RVE1 (REVEILLE 1), through transcriptomic method [65]. However, the effect of CDK8 and CYCC1 on flowering has not been reported in *Arabidopsis* until recently [64].

## 5. Perspectives

The proper timing of developmental transition is important for reproductive success in plants. Changes in the relative timing of vegetative and reproductive development have major effects on traits, including biomass, yield, secondary metabolites, and resistance to biotic and abiotic stresses. Studies have shown that nearly half of Mediator subunits have function in vegetative phase change or floral transition in *Arabidopsis*. However, of the middle module subunits, only OsMED4 has been studied regarding regulating developmental transition. Whether other middle module subunits function in this process needs to be explored. However, most crop plants have highly duplicated genomes, which implies more difficulty for further study in crops.

MED25 and MED18 have the different expression patterns and the different interacting proteins. They both positively control the floral transition, while they have opposite effects on the accumulation of plant viruses [66]. The expression level of Mediator subunits determines the structure and function of the Mediator complex. The expression of some Mediator subunits has spatial and temporal expression patterns; therefore, the specific function of the Mediator complex and their highly connected subunits need to be investigated to a deeper level. Moreover, the structural comparison of the Mediator complex at different stages or conditions of plant growth and development need to be explored.

The functional study of many Mediator subunits is mainly based on genetics and gene expression data. Only limited Mediator subunit-interacting transcription factors have been identified thus far. Therefore, more Mediator-interacting proteins should be identified to understand how they work to regulate transcription. Moreover, almost all Mediator subunits are identified and studied in *Arabidopsis*, but the functional study of their counterparts in crop plants is rare. In addition, AtMED15 in Arabidopsis function in the regulation of floral transition, whereas OsMED15 governs seed size in rice by interacting with OsNAC024 and OsNAC025 transcription factors to regulate the expression of *GW2* (*Grain Width 2*), *GW5* and *D11* (*DWARF11*) [67]. The specialized function of the Mediator complex in different plants may be attributable to the difference in interaction map of Mediator subunits. Therefore, it will be critical to purify new Mediator subunits to construct an interaction map of Mediator subunits in crop plants to reveal their function to facilitate molecular breeding in crop plants.

## Figures and Tables

**Table 1:** The function of Mediators in developmental transitions.

Mediator Submodule	Subunit	Gene Names	Functions	Interacting Proteins	Regulated Genes
Head	MED8	AT2G03070, SETH10	Floral transition		*FT*, *FLC*
	MED17	AT5G20170	Vegetative phase change and floral transition		miR159
	MED18	AT2G22370	Vegetative phase change and floral transition	SUF4, SPL15	miR159, *FLC*, *FUL*, *FT*
	MED20a	AT2G28230	Floral transition		miR172
	MED30	AT5G63480	Floral transition		*SPL3*, *FT*, *SOC1*, *FLC*
Middle	MED4	AT5G02850	Vegetative phase change	SAD1 (RPA34.5)	miR156, miR172 (in rice)
Tail	MED2	AT1G11760	Floral transition		*FLC*
	MED5	REF4	Floral transition		JA-pathway
	MED15	AT1G15780, NRB4	Floral transition		
	MED16	AT4G04920, SFR6,YID1, IEN1	Vegetative phase change and floral transition		*CCA1*, *GI*, *TOC1*, *CO*, *FT*, *FLC*
	MED23	At1g23230	Floral transition		*AG*
	MED25	AT1G25540, PFT1	Floral transition	COP1, HY5; SPL10; MBR1, MBR2; DREB2A	*CO*, *FT*, *FLC*, *FUL*
CDK8	MED12	AT4G00450, CCT, CRP	Vegetative phase change and floral transition		miR156, miR172, *FT*, *TSF*, *FLC* *SOC1*, *FUL*
	MED13	AT1G55325, GCT, MAB2	Vegetative phase change and floral transition		miR156, miR172, *FT*, *TSF*, *FLC* *SOC1*, *FUL*
	CDK8	At5g63610, HEN3	Floral transition		*FT*, *TFL1* (in pea)
	CYCC1;1/CYCC1;2	At5g48640/At5g48630	Floral transition		*FT*, *TFL1* (in pea)

## Supplementary Materials

Supplementary materials can be found at https://www.mdpi.com/1422-0067/21/8/2733/s1. Table S1 Mediator subunits are conserved within the crop plants.
