# Supplementary material for: The Important Function of Mediator Complex in Controlling the Developmental Transitions in Plants
Source: Int J Mol Sci. 2020 Apr 15;21(8):2733. doi: 10.3390/ijms21082733 (PMC7215822; doi:10.3390/ijms21082733)
Supplement: Supplementary file 1 [file ijms-21-02733-s001.pdf]

Table S1 Mediator subunits are conserved within the crop plants.

Module	Subunit	*A. thaliana*	L (aa)	Mw	pI	Domains	*G. max* (Identity %)	*Z. mays* (Identity %)	*O. sativa* (Identity %)
**Head**	**MED6**	AT3G21350	298	33411	4.7	PF04934; PTHR13104; PIRSF023869;	Glyma08g21290 (80.7)	GRMZM2G017537 (66.7)	Os06g11370 (43.4)
						G3DSA:3.10.450.580			
	**MED8**	AT2G03070	524	58157	9.97	PTHR35552	Glyma13g45020 (80.5)	GRMZM2G168913 (49.7)	Os03g31010 (35.7)
	**MED11**	AT3G01435	115	13322	4.78	PF10280; PTHR22890	Glyma03g32550 (66.1)	GRMZM2G071626 (51.8)	Os02g09600 (67.9)
							Glyma19g35290 (66.7)	GRMZM2G145996 (61.2)	Os02g49640 (52.7)
	**MED17**	AT5G20170	653	73404	6.32	PF10156; PTHR13114	Glyma05g08320 (42.4)	GRMZM2G129806 (34.4)	Os12g44140 (41.0)
							Glyma14g01070 (54.1)		
							Glyma17g12660 (42.4)		
	**MED18**	AT2G22370	219	23365	6.5	PTHR13321	Glyma09g06000 (79.8)	GRMZM2G090480 (48.1)	Os02g10050 (54.0)
							Glyma15g17250 (64.7)		
							Glyma18g18520 (63.3)		
	**MED19**	AT5G12230	221	25697	9.96	PTHR22536	Glyma04g00310 (61.0)	GRMZM2G063688 (58.0)	Os03g44010 (59.8)
		AT5G19480	207	23824	10.09	PTHR22536	Glyma06g00360 (61.0)	GRMZM2G119339 (58.3)	Os12g41962 (58.3)
							Glyma07g06450 (69.8)	GRMZM2G327174 (60.0)	
							Glyma16g03060 (69.8)		
	**MED20**	AT2G28230	219	25127	8.38	PF08612; PTHR12465	Glyma13g16910 (76.7)	AC203031.3_FGP002 (44.2)	Os09g27140 (43.1)
		AT4G09070	219	25123	5.78	PF08612; PTHR12465	Glyma17g05810 (60.2)	GRMZM2G126131 (53.2)	
		AT2G28020	70	7870	6.52	PTHR12465			
	**MED22**	AT1G07950	154	16563	4.97	PTHR12434; PF06179;	Glyma08g17030 (78.6)	GRMZM2G153766 (66.7)	Os01g04110 (60.5)
						G3DSA:1.20.58.1600	Glyma15g33520 (74.0)		Os08g37480 (61.8)
		AT1G16430	150	16179	5.22	PTHR12434; PF06179;			
						G3DSA:1.20.58.1600			
	**MED28**	AT3G52860	156	17961	6.04	PTHR39117	Glyma05g31930 (43.1)	GRMZM2G130010 (66.7)	Os05g06290 (74.4)
							Glyma08g15230 (63.4)		
	**MED30**	AT5G63480	189	19814	4.34	PTHR36406	Glyma04g37350 (47.3)	GRMZM2G143242 (41.4)	Os04g31910 (74.3)
							Glyma06g17740 (48.2)	GRMZM2G175188 (40.9)	
**Middle**	**MED1**	AT2G15890	203	23154	5.65	PTHR36345	Glyma07g20870 (54.6)	GRMZM2G009958 (36.8)	Os03g55670 (35.2)
	**MED4**	AT5G02850	426	46155	4.64	PF10018; PTHR13208	Glyma06g20060 (62.6)	GRMZM2G331754 (52.5)	Os09g36890 (54.3)
							Glyma08g39660 (64.5)	GRMZM2G452067 (52.1)	Os11g05150 (64.0)
							Glyma13g04800 (62.9)		
	**MED7**	AT5G03220	168	19425	6.36	PF05983; PTHR21428; SSF140718	Glyma07g03300 (54.8)	GRMZM2G061672 (58.2)	Os04g56640 (75.8)
		AT5G03500	168	19299	6.02	PF05983; PTHR21428; SSF140718	Glyma08g22810 (72.7)	GRMZM2G066981 (79.3)	
	**MED9**	AT1G55080	244	28586	5.95	PTHR37188	Glyma07g01150 (62.1)	GRMZM2G160527 (68.2)	Os01g31629 (63)
							Glyma08g20540 (58.6)		
	**MED10**	AT1G26665	189	20912	5.06	PTHR13345; PF09748	Glyma08g40780 (80.6)	GRMZM2G110500 (50.5)	Os09g35920 (52.5)
		AT5G41910	186	20726	5.35	PTHR13345; PF09748	Glyma18g16280 (53.2)	GRMZM2G034260 (86.5)	
	**MED14**	AT3G04740	1703	185507	7.43	PTHR12809; PF08638	Glyma03g40750 (69.4)	GRMZM2G446872 (56.2)	Os08g24400 (61.8)
							Glyma19g43440 (70.6)		Os09g10960 (55.2)
	**MED21**	AT4G04780	139	15041	4.14	PTHR13381; PF11221; 140718;	Glyma10g41060 (60.5)	GRMZM2G074280 (60.5)	Os08g04150 (73.7)
						G3DSA:1.20.58.470; SSF140718			
	**MED26**	AT3G10820	580	65817	6.95	SM00509; PTHR15141;	Glyma04g39470 (32.1)	AC194104.3_FGP007 (51.0)	Os10g10270 (33.3)
						G3DSA:1.25.40.260; PF08711;	Glyma06g15430 (32.1)	GRMZM2G153409 (45.8)	Os03g24950 (50.0)

						PS51319; SSF47676; CD00183;	Glyma19g127800 (59.7)	GRMZM2G180033 (47.9)	Os05g34210 (46.7)
						G3DSA:1.20.930.10	Glyma03g124700 (60.6)	GRMZM2G702950 (44.9)	
		AT5G05140	436	49135	7.07	SM00509; PTHR15141;	Glyma02g157800 (67.2)	AC218932.3_FGP009 (37.5)	
						G3DSA:1.25.40.260; PF08711;			
						PS51319; SSF47676; CD00183;			
						G3DSA:1.20.930.10			
		AT5G09850	353	40058	4.93	SM00509; PTHR15141;			
						G3DSA:1.25.40.260; PF08711;			
						PS51319; SSF47676; CD00183;			
						G3DSA:1.20.930.10			
	**MED31**	AT5G19910	226	26604	9.45		Glyma10g38750 (100)	GRMZM2G139683 (93.8)	Os07g07020 (93.8)
							Glyma20g28990 (100)		Os10g41450 (57.7)
**Tail**	**MED2/29/32**	AT1G11760	151	16013	4.45	PTHR21528; PTHR35989	Glyma04g14710 (65.8)	GRMZM2G441565 (58.4)	Os10g40070 (47.3)
							Glyma09g34810 (65.8)		
	**MED3/27**	AT3G09180	402	44510	8.15	PF11571; PTHR13130	Glyma03g36030 (51.6)	GRMZM2G071319 (50.0)	Os01g15850 (45.2)
							Glyma19g03150 (69.9)		
	**MED5/24/33**	AT2G48110	1275	139562	6.48	PTHR33739	Glyma03g30760 (51.4)	GRMZM2G106790 (56.7)	Os05g24684 (33.8)
		AT3G23590	1309	143093	7.11	PTHR33739	Glyma05g09220 (59.7)	GRMZM2G150754 (40.9)	Os07g11000 (55.2)
							Glyma13g28070 (67.2)	GRMZM2G151983 (34.2)	Os07g48350 (37.2)
							Glyma15g10970 (66.4)	GRMZM2G358491 (40.0)	
							Glyma18g45100 (38.5)		
							Glyma19g00780 (58.5)		
	**MED15**	AT1G15770	293	32657	9.3	PTHR33137	Glyma08g22950 (66.1)	GRMZM2G020920 (64.7)	Os04g03860 (26.9)
		AT1G15780	1335	146345	9.72	PTHR33137; G3DSA:1.10.246.20;	Glyma13g44470 (70.4)	GRMZM2G164878 (70.6)	Os08g45080 (65.4)
						PF16987	Glyma15g00790 (67.8)		
		AT2G10440	935	105506	8.99	PTHR33137; PF16987			
	**MED16**	AT4G04920	1278	138185	6.13	PTHR35130	Glyma13g24970 (73.4)	GRMZM2G119657 (65.6)	Os10g35560 (61.9)
							Glyma13g31480 (72.2)		
							Glyma15g07830 (68.6)		
	**MED23**	AT1G23230	1615	180094	7.36	PTHR12691; PF11537	Glyma09g22260 (71.4)	GRMZM2G089684 (59.9)	Os02g49992 (50.0)
	**MED25**	AT1G25540	836	89566	8.34	PTHR10270; SM00327(VWF_A);	Glyma02g10880 (54.5)	GRMZM2G138178 (36.8)	Os09g13610 (82.8)
						PF11265; PTHR12433			
**Kinase**	**MED12**	AT4G00450	2235	247224	8.93		Glyma03g01150 (59.3)	GRMZM2G114459 (43.2)	Os07g45400 (46.1)
							Glyma07g07710 (50.6)	GRMZM5G828278 (43.4)	Os10g40260 (37.9)
							Glyma09g39550 (59.6)		
							Glyma18g46700 (58.3)		
	**MED13**	AT1G55325	2001	217267	6.19		Glyma18g00280 (63.7)	GRMZM2G153792 (52.2)	Os05g37500 (51.8)
							Glyma11g256800 (64.0)		
	**CDK8**	AT5G63610	470	52797	9.62	SM00220; PS50011; PS00108;	Glyma04g38510 (40.4)	GRMZM2G166771 (32.0)	Os10g42950 (48.1)
						PF00069; SSF56112; PS00107;	Glyma08g00510 (80.0)		
						G3DSA:1.10.510.10;	Glyma08g10810 (40.7)		
						PTHR24056:SF92; PTHR11295:SF83;			
						PTHR11295; 56112; PTHR24056;			
						CD07842; G3DSA:3.30.200.20			

	**CYCC1**	AT5G48630	256	30214	6.91	PIRSF028758; PF00134; SSF47954; SM00385; G3DSA:1.10.472.10; PTHR10026:SF7; PTHR10026; 47954; CD00043	Glyma08g46170 (50.8)	GRMZM2G408242 (70.7)	Os09g32680 (77.5)
		AT5G48640	253	29914	6.43	SM00385; SSF47954; G3DSA:1.10.472.10; PIRSF028758; PF00134; 47954; PTHR10026; PTHR10026:SF7; CD00043	Glyma18g33140 (52.4)		
**Unknown**	**MED34**	AT1G31360	705	79368	7.49	PF00271; TIGR00614; SM00490; PF00270; SM00487; PF00570; PTHR13710; PS50967; PS51192; PF09382; G3DSA:3.40.50.300; SSF52540; PS51194; PTHR13710:SF12; PTHR13710:SF72; CD00046; CD00079; G3DSA:1.10.10.10; G3DSA:1.10.150.80; PF16124; SSF47819; SM00956	Glyma09g34860 (62.5)	GRMZM2G451856 (43.1)	Os04g35420 (40.7)<br>Os11g48090 (78.9)
	**MED35**	AT1G44910	958	109381	6.43	PF00397; PS50020; SM00456; SM00441; PF01846; SSF51045; PTHR11864; SSF81698; G3DSA:1.10.10.440; G3DSA:2.20.70.10; PTHR11864:SF21; CD00201; PS51676	Glyma10g255700 (42.2)<br>GlymaU029000 (68.3)<br>Glyma17g35120 (73.2)<br>Glyma20g27240 (59.4)		Os01g34780 (64.3)
		AT3G19670	992	113568	6.91	PF00397; PS50020; SM00456; SM00441; PF01846; SSF51045; PTHR11864; SSF81698; G3DSA:1.10.10.440; G3DSA:2.20.70.10; PTHR11864:SF21; CD00201; PS51676			
		AT3G19840	835	92806	8.68	SM00441; SSF81698; PF00397; PS01159; PS50020; SSF51045; SM00456; PF01846; PTHR15377; G3DSA:2.20.70.10; G3DSA:1.10.10.440			
	**MED36**	AT4G25630	320	33653	10.69	PF01269; PR00052; PTHR10335; PIRSF006540; SSF53335 G3DSA:3.40.50.150; SM01206; G3DSA:3.30.200.20; MF_00351	Glyma04g37770 (84.7)<br>Glyma11g237200 (77.2)<br>Glyma06g17310 (85.5)<br>Glyma18g02340 (67.3)	GRMZM2G150648 (94.9)<br>GRMZM2G105480 (51.5)	Os02g57590 (96.6)<br>Os05g08360 (64.3)
		AT5G52470	308	32829	10.8	PF01269; PR00052; PTHR10335; PIRSF006540; SSF53335 G3DSA:3.40.50.150;			

					G3DSA:3.30.200.20; MF_00351; SM01206			
**MED37**	AT1G09080	675	75149	4.68	PTHR19375; PF00012; SSF100920; PS00297; G3DSA:3.90.640.10; SSF53067; PS01036; PS00329; G3DSA:3.30.420.40; PR00301; SSF100934; G3DSA:2.60.34.10; PTHR19375:SF1	Glyma02g10320 (63.9)<br>Glyma03g32850 (60.8)<br>Glyma15g 088000 (56.7)<br>Glyma13g 130900 (63.4)<br>Glyma05g36600 (64.7)<br>Glyma17g 072400 (63.9)	AC209784.3_FGP007 (63.9)<br>GRMZM2G415007 (69.4)<br>GRMZM2G428391 (79.4)<br>GRMZM2G310431 (63.1)<br>GRMZM2G340251 (61.5)<br>GRMZM2G056039 (63.0)	Os01g62290 (62.6)<br>Os02g02410 (50.6)<br>Os03g16860 (62.6)<br>Os05g23740 (63.0)<br>Os09g31486 (50.4)<br>Os12g38180 (66.0)
	AT3G12580	650	71101	4.88	PS00297; PS00329; PR00301; PF00012; PS01036; PTHR19375; SSF100934; PTHR19375:SF1; G3DSA:3.90.640.10; SSF53067; SSF53067; G3DSA:2.60.34.10; SSF100920; CD10233; G3DSA:3.30.420.40; PTHR19375:SF323; G3DSA:1.20.1270.10; G3DSA:3.30.30.30	Glyma18g52610 (61.9)<br>Glyma08g02940 (71.0)<br>Glyma08g02960 (78.7)<br>Glyma11g14950 (61.7)<br>Glyma12g06910 (61.7)<br>Glyma07g26550 (50.6)<br>Glyma19g35560 (61.1)		Os08g09770 (70.9)<br>Os11g47760 (61.7)
	AT5G02490	653	71386	4.75	PR00301; PS00297; PS01036; PF00012; PS00329; PTHR19375; PTHR19375:SF1; G3DSA:3.90.640.10; G3DSA:3.30.420.40; SSF100934; SSF100920; G3DSA:2.60.34.10; SSF53067; 100920; 53067; 100934; CD10233; PTHR19375:SF323; G3DSA:1.20.1270.10; G3DSA:3.30.30.30			
	AT5G02500	651	71357	4.75	PS01036; PS00297; PS00329; PR00301; PR00301; PF00012; PTHR19375; G3DSA:3.90.640.10; PTHR19375:SF1; G3DSA:2.60.34.10; SSF100920; G3DSA:3.30.420.40; SSF100934; SSF53067; 53067; 100934; 100920; PTHR19375:SF323; CD10233; G3DSA:1.20.1270.10; G3DSA:3.30.30.30			
	AT5G28540	669	73629	4.81	PF00012; PS00329; PS01036; PS00297; PR00301; PTHR19375; PS51257; G3DSA:2.60.34.10; SSF100920; SSF53067; SSF100934; G3DSA:3.30.420.40; G3DSA:3.90.640.10; PTHR19375:SF1; 100934; 53067;			

					100920; PTHR19375:SF317; CD10241; G3DSA:1.20.1270.10
	AT5G42020	668	73561	4.84	PS01036; PR00301; PS00297; PF00012; PS00329; PTHR19375; PS51257; PTHR19375:SF1; SSF53067; G3DSA:3.90.640.10; SSF100920; SSF100934; G3DSA:2.60.34.10; G3DSA:3.30.420.40; 53067; 100920; 100934; PTHR19375:SF317; CD10241; G3DSA:1.20.1270.10;

The numbers within the brackets indicate sequence identities (%) between Arabidopsis Mediator subunits and their orthologues in other species. The source databases of *A. thaliana*, *G. max*, *Z. mays* and *O. sativa* are from *Arabidopsis thaliana TAIR10* (https://www.arabidopsis.org), *Glycine max Wm82.a2.v1* (https://phytozome.jgi.doe.gov), *Oryza sativa V7_JGI* (https://phytozome.jgi.doe.gov) and *Zea mays Ensembl_18* (https://phytozome.jgi.doe.gov), respectively. The Arabidopsis Mediator sequences were downloaded from TAIR10 (https://www.arabidopsis.org) by name search; and their homologs were identified by BLAST in this database. For identifying Mediator homologs in *G. max*, *Z. mays* and *O. sativa*, BLAST searches were performed based on the seed sequences from *Arabidopsis* using the BLOSUM 62 substitution matrix and an iteration threshold of 0.1. The proteins with the presence of common MED domains were selected. ~~All the data are from websites, https://www.arabidopsis.org and https://phytozome.jgi.doe.gov.~~

L, protein length; Mw, Molecular weight; pI, Isoelectric points.

Formatted: Font: Italic
Formatted: Font: Italic
Formatted: Font: Italic
Formatted: Font: Italic
Formatted: Font: Italic
Formatted: Default Paragraph Font
Formatted: Font: Italic
Formatted: Default Paragraph Font
Formatted: Font: Italic
Formatted: Default Paragraph Font
Formatted: Font: Italic
Formatted: Default Paragraph Font
Formatted: Default Paragraph Font
Formatted: Font: Italic
